# Multi‐omics profiling of chronic immune‐mediated skin diseases: SKINERGY protocol and strategic evaluation

**DOI:** 10.1111/jdv.70311

**Published:** 2026-02-06

**Authors:** N. G. Koster, J. M. P. A. van den Reek, E. M. G. J. de Jong, S. van Beugen, A. I. M. van Laarhoven, I. Haeck, M. de Bruin‐Weller, M. L. Schuttelaar, B. Horvath, A. Gostyński, A. Knulst, M. H. Vermeer, L. Nguyen, L. Gerbens, P. Spuls, M. A. Middelkamp‐Hup, V. Exadaktylos, T. Niemeyer‐van der Kolk, A. R. Schrader, A. M. Sluijmers, J. Versteeg, B. W. M. Arents, I. van Ee, J. L. A. G. van der Zon, M. de Leeuw, T. de Leeuw, P. van den Broek, C. Berkhof, D. Vellinga, S. Weidinger, S. Dubrac, Y. Li, M. van Steensel, M. Marchetti‐Deschmann, M. Maurer, H. van der Zee, J. Damman, D. J. Hijnen, F. Wijk, M. M. B. Seyger, H. Röckmann, D. M. W. Balak, M. B. A. van Doorn, R. Rissmann, The Next Generation ImmunoDermatology (NGID) consortium, Rob Vreeken, Eva Cuypers, Boudewijn Lelieveldt, Abdoel El Ghalbzouri, Hanna Niehues, Ellen van den Bogaard, Tom Ederveen, Els van der Pool, Nanda Olde, Debra Trampe, Ellen Stijl‐’t Hart, Lisanne Wezendonk, Johan Westerhuis, Huma Shehwana, Joerg Schreiber, Jeremy Bost, Mie Bech Lukassen, Jimmy Bakker, Juliette Simons, Fauve van den Berge, Colin Spence, Elise Beljaards, Anastasiia Myronenko, Mariona Oliver

**Affiliations:** ^1^ Division BioTherapeutics, Leiden Academic Centre for Drug Research Leiden University Leiden The Netherlands; ^2^ Department of Dermatology Leiden University Medical Center Leiden The Netherlands; ^3^ Centre for Human Drug Research (CHDR) Leiden The Netherlands; ^4^ Dept. Dermatology Radboud University Medical Centre Nijmegen The Netherlands; ^5^ Leiden University, Institute of Psychology, Health, Medical and Neuropsychology Unit Leiden The Netherlands; ^6^ Department of Dermatology University Medical Center Utrecht Utrecht The Netherlands; ^7^ Department of Dermatology University Medical Center Groningen Groningen the Netherlands; ^8^ Department of Dermatology University Medical Center Maastricht+ Maastricht the Netherlands; ^9^ GROW Research Institute for Oncology and Reproduction, Maastricht University Maastricht The Netherlands; ^10^ Department of Dermatology Amsterdam Institute for Public Health, Immunology and Infectious Diseases, Amsterdam UMC, Location Academic Medical Center, University of Amsterdam Amsterdam The Netherlands; ^11^ University Medical Center Utrecht, Center of Translational Immunology Utrecht the Netherlands; ^12^ Department of Pathology Leiden University Medical Center Leiden the Netherlands; ^13^ Dutch Association for People with Lupus, APS, Scleroderma and MCTD Utrecht the Netherlands; ^14^ Dutch Association for Patients with Hidradenitis Utrecht The Netherlands; ^15^ Dutch Association for People with Atopic Dermatitis Nijkerk the Netherlands; ^16^ Dutch Association for People with Psoriastic Disease Nijkerk the Netherlands; ^17^ Dutch Cutaneous Lymphoma Patient Advocacy Group Utrecht The Netherlands; ^18^ Dutch Skin Alliance Utrecht The Netherlands; ^19^ Dutch Patient Platform Advocacy Group Urticaria Utrecht The Netherlands; ^20^ Alrijne Hospital Leiderdorp Leiderdorp The Netherlands; ^21^ University Hospital Kiel Schleswig Holstein Kiel Germany; ^22^ Medical University of Innsbruck Innsbruck Austria; ^23^ Helmholtz Centre for Infection Research, Hannover Medical School Hannover Germany; ^24^ Nanyang Technological University Singapore; ^25^ Technical University Wien Vienna Austria; ^26^ Charité University Hospital Berlin Germany; ^27^ Department of Dermatology Erasmus MC, University Medical Center Rotterdam Rotterdam The Netherlands

**Keywords:** atopic dermatitis, chronic spontaneous urticaria, cutaneous lupus erythematosus, hidradenitis suppurativa, inflammatory skin diseases, multi‐omics deep phenotyping, mycosis fungoides‐type cutaneous t‐cell lymphoma, personalized medicine, psoriasis

## Abstract

**Background:**

The Dutch flagship project Next Generation ImmunoDermatology (NGID) aims to profile five chronic immune‐mediated inflammatory skin diseases: atopic dermatitis (AD), plaque psoriasis (PSO), hidradenitis suppurativa (HS), chronic spontaneous urticaria (CSU) and cutaneous lupus erythematosus (CLE) in comparison with cutaneous T‐cell lymphoma subtype mycosis fungoides (MF) and healthy volunteers. Within NGID, a clinical study entitled: ‘SKIN disease profiling by an Exploratory, pRospective, biomarker study in dermatoloGY practice (SKINERGY)’ will be conducted as a multicentre, parallel‐cohort, open‐label, observational, longitudinal basket study.

**Objectives:**

Objectives include evaluation of disease‐related characteristics in comparison to those of healthy volunteers and evaluation of biomarkers for disease stratification and (targeted) treatment response in patients in a real‐world clinical setting. Additionally, differences and similarities in disease characteristics between diseases, changes over time, and profiles of responders versus non‐responders will be evaluated.

**Methods:**

Patients with AD (*N* = 120), PSO (*N* = 160), HS (*N* = 80), CSU (N = 120) and CLE (N = 120) will be enrolled in groups of *N* ≤ 40 patients per treatment. Matched healthy volunteers (N = 120) and the MF cohort (N = 120) will serve as control groups. Assessments include blood sampling, skin punch biopsies, tape stripping, skin swabs, (multimodal) imaging, tele‐health and patient‐ and physician‐reported outcomes. This manuscript describes the study protocol prior to data collection and its strategic evaluation of multi‐omics profiling. Patient advocacy groups co‐defined the research agenda and contributed to study design and informed consent document development, ensuring alignment with patients' needs and real‐world relevance.

**Results:**

SKINERGY will generate a machine learning‐ready dataset with information about changes in various biomarkers over time, including histology, metabolomics, spatial proteomics, transcriptomics, lipidomics, microbiomics, imaging biomarkers, tele‐health, patient‐reported outcome measures (PROMs) and clinical parameters.

**Conclusions:**

Identified biomarker profiles within SKINERGY may guide targeted treatment selection, enhance targeted therapeutic response in clinical practice and improve understanding of disease pathology in chronic immune‐mediated skin diseases.

## Plain Language Summary

The SKINERGY study will investigate disease mechanisms of five immune‐mediated inflammatory skin diseases, namely atopic dermatitis, psoriasis, hidradenitis suppurativa, chronic spontaneous urticaria and cutaneous lupus erythematosus. Chronic inflammatory skin conditions are estimated to affect up to 25% of the population. The study is designed to compare mechanisms of these inflammatory skin diseases with an external cohort of patients with cutaneous T‐cell lymphoma subtype mycosis fungoides and healthy volunteers.

The SKINERGY study is a nationwide initiative, with patient inclusion occurring across all university medical centres in the Netherlands.

The main aim of the SKINERGY study is to generate a harmonized dataset suitable for advanced computer analysis in order to identify biomarkers for disease monitoring and enabling prediction of (non)‐response to treatment.

This is an observational study, in which 720 patients will be followed for 1 year after initiating real‐world treatment in routine clinical settings. Several parameters will be assessed at baseline (prior to the first dose), Month 3 and Month 6 (core study). Optional assessments include a 12‐month follow‐up visit, supplementary questionnaires and skin punch biopsies. Additionally, a control group of 120 untreated healthy volunteers will be monitored over a 3‐month period.

Ultimately, the study seeks to discover biomarkers that can guide treatment selection, facilitating more precise and personalized treatment strategies for patients with inflammatory skin diseases.

## Graphical abstract

The SKINERGY study will investigate six immune‐mediated inflammatory skin diseases through a nationwide collaboration across all Dutch university medical centres. Involving 720 patients and 120 healthy volunteers, it is aimed at generating a high‐quality dataset to identify biomarkers that enable personalized treatment strategies and improved disease monitoring in clinical practice.

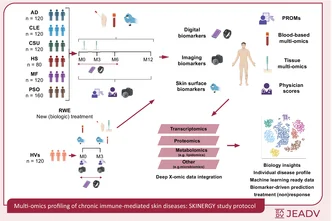

**Why was the study undertaken?**

This study aims to address the limited yield of previous biomarker research in chronic immune‐mediated inflammatory skin diseases by taking a comprehensive, real‐world approach. We will investigate how disease‐related characteristics and biomarker profiles differ among patients with these diseases, how they change over time and how they relate to treatment response. Comparisons with patients with MF and healthy volunteers will serve as a reference to better understand disease‐specific patterns.

**What does this study add?**

SKINERGY aims to provide real‐world, cross‐disease, longitudinal insights into chronic immune‐mediated inflammatory skin diseases. By integrating multi‐omics profiling, spatial tissue analysis and patient‐ and physician‐reported outcomes across six diseases and healthy controls, the study is expected to identify both shared and disease‐specific mechanisms. Its nationwide, representative design and mechanism‐based patient stratification may generate a harmonized, high‐quality dataset. This dataset will support machine learning approaches for biomarker identification and validation.

**What are the implications of this study for disease understanding and/or clinical care?**

The SKINERGY study is designed to advance the understanding of immune‐mediated inflammatory skin diseases. This will be done through repetitive multi‐omics profiling, with a real‐world study design. By linking biomarker profiles to treatment response, disease characteristics and disease endotypes, SKINERGY has the potential to support the development of personalized treatment algorithms in routine dermatological care.

## INTRODUCTION

Immune‐mediated inflammatory skin diseases are highly prevalent, affecting an estimated 20%–25% of the population,^1^ and have a major physical and psychological impact. Patients often experience persistent itch, sleeplessness, low self‐esteem and, ultimately, a critical impact on their participation in society.^2^ The long‐term impact of inflammatory skin disorders is debilitating as direct consequences span from a lifelong negative impact on quality of life (QoL), decreased productivity at work^3^ and psychiatric comorbidities to a high risk of cardiovascular and metabolic complications.^4^, ^5^, ^6^ The significant burden on patients underscores the need for improved dermatological treatments.^6^, ^7^, ^8^, ^9^ Additionally, generic treatment protocols, that is, ‘one size fits all’, with costly targeted drugs, that is, focusing on a single, distinct molecular target, lead to very high and ever‐increasing healthcare costs in dermatology. This highlights the need for more efficient, tailored strategies, as such broad approaches overlook the complexity of disease transitions.^10^

Numerous studies have highlighted the role of deep phenotyping and disease characterization in enhancing personalized healthcare.^11^, ^12^, ^13^, ^14^, ^15^ Researchers have emphasized the promising value of therapeutic matching using patient biopsies prior to initiating targeted treatments.^16^ Additionally, single‐cell profiling with omics techniques offers great potential for deep phenotyping and a more comprehensive understanding of disease characteristics and endotypes.^17^, ^18^, ^19^ Moreover, microbial communities influence immune balance, drive inflammation and contribute to the progression of inflammatory skin diseases.^20^ Implementing these deep phenotyping approaches is crucial for enhancing personalized treatment in immune‐mediated inflammatory skin diseases, with resulting biomarkers serving as indicators of underlying biological and pathogenic processes, as well as treatment responses.

Currently, various international consortia and research groups are gathering clinical data for deep phenotyping purposes using multi‐omics approaches in different cohorts, for example, the ImmUniverse consortium, the Biomarkers in Atopic Dermatitis and Psoriasis project (BIOMAP), the HIPPOCRATES research programme and the Translational Dermatology Initiative.^21^, ^22^, ^23^, ^24^

Our SKIN disease profiling by an Exploratory, pRospective, biomarker study in dermatoloGY practice (SKINERGY) study is embarked on to further investigate disease mechanisms of atopic dermatitis (AD), psoriasis (PSO), hidradenitis suppurativa (HS), chronic spontaneous urticaria (CSU) and cutaneous lupus erythematosus (CLE) in comparison with cutaneous T‐cell lymphoma subtype mycosis fungoides (MF) and healthy volunteers (HV). The main aim of this observational, longitudinal biomarker study in a real‐world daily practice setting is to generate a harmonized, nation‐wide, high‐quality dataset suitable for machine learning. This may enable the identification of discriminative biomarkers for disease monitoring and identification of patterns of (non)‐response to treatment in The Netherlands. Biomarkers identified in this study require subsequent validation to ensure their reliability, in accordance with FDA guidelines.^25^, ^26^, ^27^ The ultimate goal is to evolve dermatological care from a one‐size‐fits‐all approach to personalized treatment. This manuscript describes the study protocol prior to data collection.

## METHODS

### Design and setting

The SKINERGY study is a national, multicentre, parallel‐cohort, open‐label, observational, longitudinal biomarker study in a real‐world daily practice setting with an observation period of 1 year. This study aims to characterize five relevant immune‐mediated inflammatory skin diseases, along with a lymphoproliferative skin disorder (MF) and HV as control cohorts. This study was initiated within the Next Generation ImmunoDermatology project and collaborates with all Dutch University Medical Centers, that is, Leiden University Medical Center (LUMC), University Medical Center Rotterdam (Erasmus MC), University Medical Center Utrecht (UMCU), Amsterdam University Medical Centers (AUMC), Maastricht University Medical Center (MUMC+), University Medical Center Groningen (UMCG), Radboud University Medical Center (RUMC) and the Centre for Human Drug Research (CHDR). In total, 720 patients will be followed for 1 year after the commencement of a real‐world treatment in the healthcare setting. Multimodal parameters will be evaluated pre‐dose (baseline), at month 3 and at month 6 (core study). The study offers optional assessments, which comprise a 12‐month follow‐up visit, supplementary questionnaires and skin punch biopsies. A total of 120 untreated healthy volunteers will be monitored over 3 months as the control group, with multimodal assessments at baseline and month 3.

### Ethical considerations

The study will be conducted in accordance with the following: (i) the principles of the Declaration of Helsinki, (ii) the Medical Research Involving Human Subjects Act (WMO), (iii) the EU's General Data Protection Regulation effective 25 May 2018 and (iv) applicable laws and regulations. All study procedures have been approved by the Medical Ethics Committee METC Leiden Den Haag Delft, the Netherlands (reference P24.100). Amendments to the study protocol will be submitted for review at the Medical Ethics Committee.

### Participants

Patients with five different inflammatory skin diseases will be enrolled: AD, PSO, HS, CSU and CLE, along with a control cohort of MF patients. Different treatment groups will be included per indication, with a minimum of *N* = 40 patients per treatment arm. In total, 120 patients (PSO = 160, HS = 80) with a broad spectrum of disease severity, that is, primarily moderate to severe, will be investigated. Participants must be 18 years or older, able to provide written informed consent, and have a confirmed diagnosis of the relevant disease. Exclusion applies if any other condition or factor could interfere with the study, as judged by the investigator. More detailed, including disease‐specific inclusion and exclusion criteria, are listed in detail in the protocol main body (Supplementary Information). Importantly, the patient must be eligible to initiate or re‐initiate a treatment that is either new to them or a restart after a protocol‐defined washout period, in agreement with the protocol and patient care setting. All healthy participants must be in stable good health as per judgement of the investigator based on medical history and assessments performed at screening. Details are provided in the protocol main body (Supplementary Information).

### Sample size

This is an exploratory, hypothesis‐generating study, and the sample size is not strictly based on a statistical rationale. The sample size of up to *N* = 120 per disease (PSO *N* = 160, HS *N* = 80) is deemed sufficient to achieve the primary aim of this study, that is, to characterise the disease in‐depth at baseline and monitoring treatment response of patients in daily practice. A total of 120 patients per disease (PSO *N* = 160, HS *N* = 80) will be enrolled, with *N* ≤ 40 patients assigned to each mechanism‐based treatment arm. Given the observational nature of the study, a sample size of N ≤ 40 patients per treatment was chosen to ensure a sufficiently large cohort, allowing for the inclusion of both super‐responders and non‐responders (expected *N* > 6–8 for almost all treatment arms). In addition, this is a longitudinal study to identify and validate biomarkers. If group differences are too small at the selected sample size, preventing statistically significant discrimination between patient subgroups (e.g. responders vs. non‐responders, lesional vs. non‐lesional skin), the biomarker would be deemed unsuitable for clinical implementation. A control group of 120 healthy volunteers will be enrolled.

### Randomization and blinding

Randomization and blinding are not applicable as it is an observational, non‐randomized, open‐label, real‐world study.

### Intervention and treatment

**FIGURE 1:**
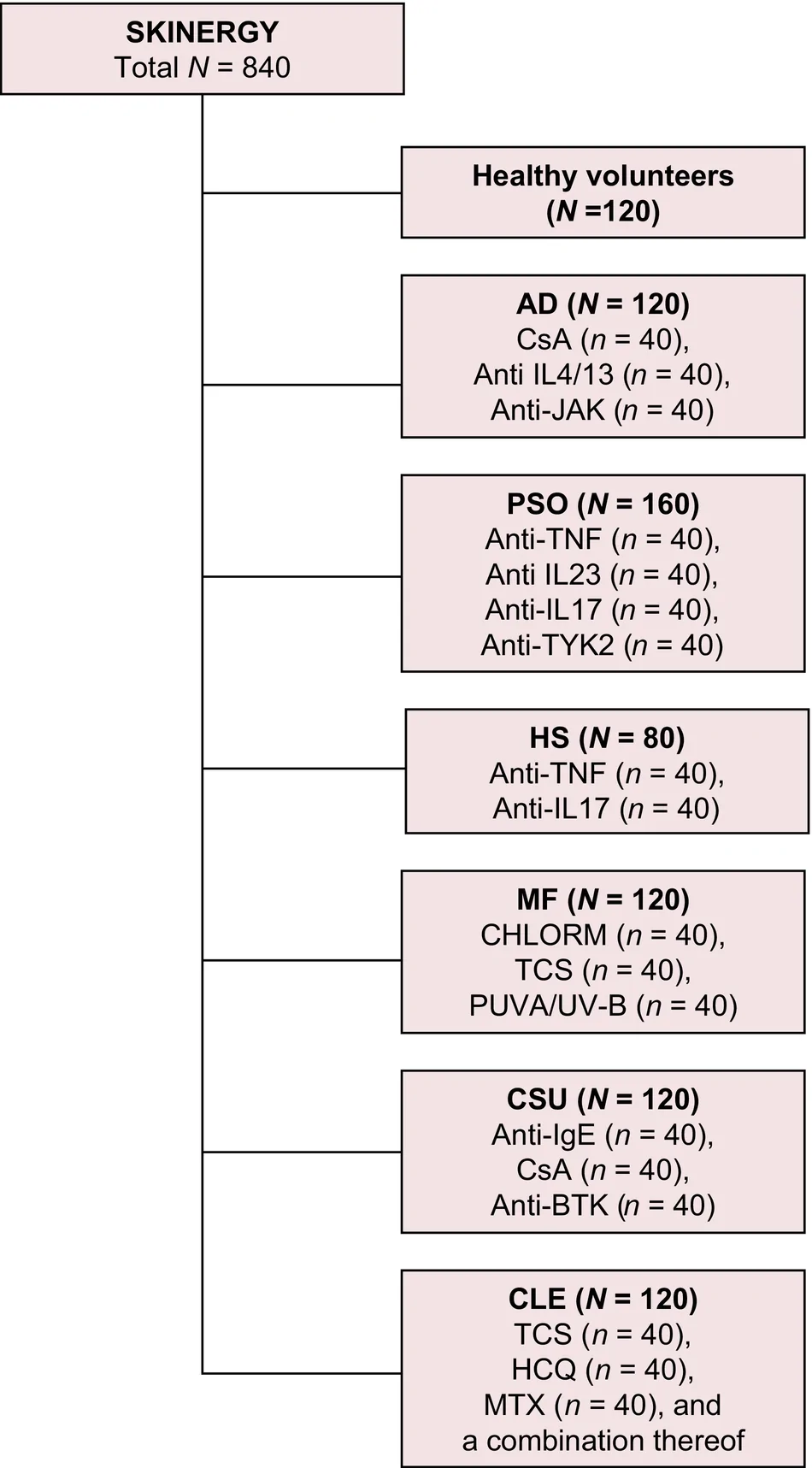
Cohorts and treatment groups for healthy volunteers, and patients with atopic dermatitis (AD), psoriasis (PSO), hidradenitis suppurativa (HS), chronic spontaneous urticaria (CSU), cutaneous lupus erythematodes (CLE) and mycosis fungoides‐type cutaneous T‐cell lymphoma (MF). Abbreviations: cyclosporine A (CsA), Anti‐Interleukin 4/13 (Anti‐IL4/13), Anti‐Janus kinase (Anti‐JAK), Anti‐tumour necrosis factor (Anti‐TNF), Anti‐interleukin 23 (Anti‐IL23), Anti‐interleukin 17 (Anti‐IL17), Anti‐tyrosine kinase 2 (Anti‐TYK2), chlormethine (CHLORM), topical corticosteroids (TCS), psoralen + ultraviolet A / ultraviolet B (PUVA/UV‐B), anti‐immunoglobulin E (Anti‐IgE), Anti‐Bruton's tyrosine kinase (Anti‐BTK), hydroxychloroquine (HCQ), methotrexate (MTX).

After enrolment in the study, all patients will initiate real‐world therapy as part of standard care according to the discretion of the attending physician. Therefore, these treatments are not considered interventions in the study. The treatment groups per disease are depicted in Figure 1.

### Primary and secondary outcomes

The primary outcomes of this study will be the percentage of patient (non)‐responders (see Appendix S1), with treatment response defined per disease‐specific criteria based on established thresholds for clinical improvement (e.g. EASI, PASI, CLASI‐A, IHS4), as well as change (Δ) in clinical parameters at 3, 6 and 12 months (Table S1), patient‐reported outcome measures, the time to switch to another therapy, skin surface proteomics and lipidomics, skin microbiome, target lesion and non‐lesional imaging and circulating biomarkers in blood.

The secondary outcomes for this study include patient‐reported outcome measures (PROMs) (Table S2), sleep quality, number of steps and heart rate, percentage of therapy failures and dropouts, type, frequency and quantities of used topical therapies, decrease in disease activity, following physician‐reported outcome measures.

Exploratory endpoints include total body photography, target lesion imaging and skin punch biopsies for histology, immunohistochemistry and multi‐omics strategies including transcriptomics (bulk RNA sequencing), metabolomics (spatial metabolomics using MALDI‐MSI and LC–MS, spatial lipidomics) and proteomics (imaging mass cytometry) and microbiome analysis. A substudy of the following technologies will be deployed: optical coherence tomography for skin morphology and epidermal thickness, multispectral imaging for 3D skin morphology, erythema, haemoglobin, melanin levels and laser speckle contrast imaging for microcirculation.

### Multi‐omics strategy

**FIGURE 2:**
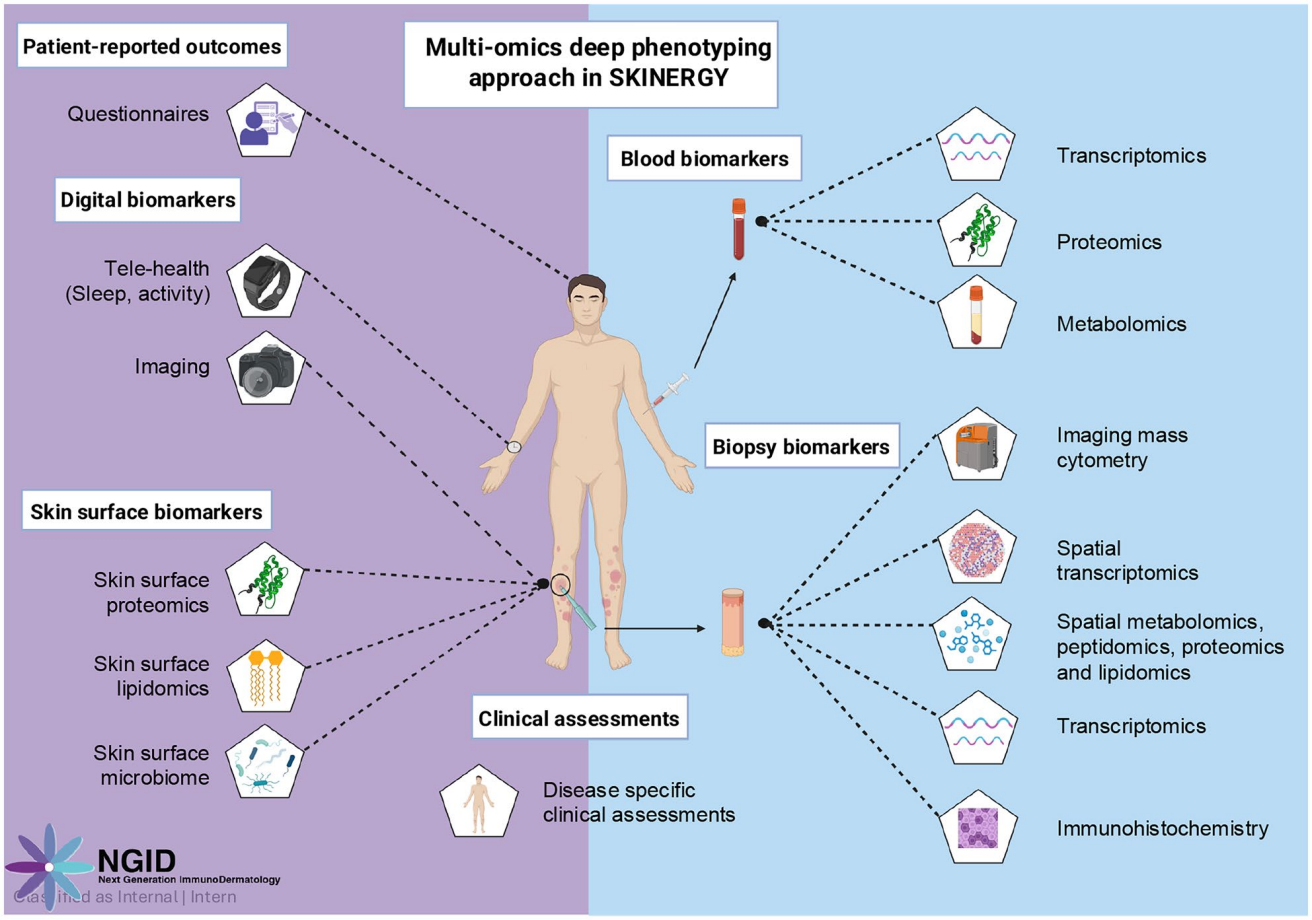
Multi‐omics strategy for deep phenotyping of inflammatory skin diseases, combining blood, skin tissue via biopsies, skin surface assessments via tape‐strips, imaging, patient‐reported outcome measures (PROMs) and wearables. Advanced techniques enable a comprehensive characterization of disease mechanisms and therapeutic responses and play a pivotal role in enabling personalized treatment strategies.

A diverse range of advanced technologies will provide a comprehensive understanding of disease mechanisms in patients with immune‐mediated inflammatory skin diseases (Table S3). Proteomics, lipidomics and microbiome analyses capture molecular and environmental factors influencing disease. Gene expression profiling using RNA sequencing will reveal the transcriptional signatures underlying disease pathogenesis in blood and skin tissue, and blood analytes provide a window into systemic biomarkers. Tissue‐level profiling will be further explored using imaging mass cytometry and spatial transcriptomics, providing a detailed view of cellular interactions and tissue architecture. Additionally, immunohistochemistry will enhance our ability to assess immune responses and tissue changes, which may provide a clearer perspective on disease progression. This integrated multi‐omics approach may not only deepen our understanding of inflammatory skin diseases but may also help us identify novel disease subtypes and lay the foundation for more targeted and personalized therapeutic strategies (Figure 2; Table 2).

### Data collection, monitoring and data analysis

Data will be entered in Castor Electronic Data Capture (EDC) and monitored by an independent monitor. Questionnaire data collected through electronic patient‐reported outcome tools is automatically stored in the Castor database. Analyses will follow an intention‐to‐treat approach. Per endpoint, descriptive statistics will quantify the response. Repeated measurements will be analysed with a mixed model analysis of variance with fixed factors group (patients and healthy controls) and the interactions and subject, subject by day and subject by time as random factors. Additionally, the contrast between responders and partial‐ or non‐responders will be assessed. The estimated intra‐subject variability over time will be assessed, and within patients, the contrast between lesional and non‐lesional skin and effects of the different treatments will be assessed. The contrast between the patients and the healthy controls will be calculated within the model. Furthermore, contrasts between patient subgroups will be explored. Exploratory and supervised multivariate (multi‐omics) analyses will be performed using multi‐omics factor analysis approaches, with all exploratory inferences treated as non‐confirmatory, while visualization and testing will be performed with high dimensional ANOVA methods. As additional time‐to‐event context, therapy switching is analysed with Kaplan–Meier and log‐rank methods.

### Patient participation

A key aim of the NGID consortium, in which the SKINERGY study is embedded, is the structural integration of patient participation. Patient organizations are actively involved in co‐defining the research agenda, prioritizing technology development and providing input on study design and implementation. Furthermore, patient organizations play a vital role in raising awareness of the study among relevant patient populations and contributing to strategies for participant inclusion. In parallel, the consortium is committed to continuously evaluating and improving the framework for patient engagement within NGID, to ensure that patient participation is systematically embedded, meaningful and impactful. Through this participatory approach, the NGID consortium seeks to ensure that the SKINERGY study is not only scientifically rigorous but also closely aligned with patients' needs, preferences and real‐world experiences.

## DISCUSSION

The SKINERGY study is a multicentre, nationwide, parallel‐cohort, open‐label, non‐randomized, observational, longitudinal biomarker study in a real‐world daily practice setting that aims to identify discriminative potential biomarkers for predictivity of treatment response in The Netherlands. This includes evaluating disease‐related (multi‐omics) biomarkers for disease stratification and targeted treatment (non‐)response over time in patients with AD, PSO, HS, CSU and CLE, compared to an MF control group and healthy volunteers. This enables comparison of inflammatory skin diseases to malignant inflammatory processes in the skin. Additionally, exploratory objectives include the identification of skin tissue and imaging biomarkers correlated with disease progression or therapy response and evaluation of patients’ genomic profiles. By identifying these potential biomarkers, we aim to shift from a ‘one‐size‐fits‐all’ approach to personalized medicine plans through a deep phenotyping approach in real‐world practice.^11^, ^16^, ^17^, ^20^

In previous studies, specific blood‐based biomarkers for inflammatory skin diseases have demonstrated to be highly effective in predicting treatment responses and enabling disease stratification. For instance, an association has been observed between pre‐treatment low serum IgE levels (<30 IU/mL) and partial slow, or non‐response to anti‐IgE therapy with omalizumab in patients with CSU.^28^, ^29^, ^30^ This biomarker is now integrated into clinical practice and can be used to counsel patients eligible for omalizumab treatment. Additionally, several biomarkers associated with the auto‐allergic CSU profile and biomarkers related to the autoimmune type IIb profiles of CSU have been identified, providing valuable insights for disease endotyping in CSU patients and therewith treatment stratification.^31^

Beyond biomarkers for CSU, predictive biomarkers for other inflammatory skin diseases, such as AD, PSO and HS, have been evaluated in previous studies. In PSO, prior research has investigated genetic polymorphisms, with particular attention to HLA‐C*06:02; however, this allele has not demonstrated sufficient predictive value for clinical response in routine practice.^32^ Literature suggests that interleukin (IL)‐12 serum levels and polymorphisms in the *IL12B* gene could serve as predictive biomarkers for treatment response in plaque psoriasis.^33^ Moreover, HCC‐4, calprotectin and fractalkine have been identified as potential predictive biomarkers for adalimumab response in patients with HS.^34^ Although these biomarkers have been identified and are linked to therapy response, they are not yet routinely applied in clinical practice. The SKINERGY study seeks to facilitate their translation into standard care and optimize individualized treatment strategies.

In addition to blood‐based biomarkers, other types of biomarkers might serve as effective predictors of treatment response or can be monitored over time to evaluate disease progression. For instance, previous research has shown that changes in the lesional ceramide profile during treatment with anti‐IL‐23 (guselkumab) correlate with barrier function and target lesion severity in patients with psoriasis (PSO).^35^, ^36^, ^37^ Furthermore, imaging techniques, such as three‐dimensional photography of target lesions, can effectively assess parameters like roughness and erythema and may offer valuable insights into disease dynamics and treatment outcomes.^38^, ^39^ Additionally, there is a substantial evidence‐base for the psychological impact of chronic skin conditions. Accumulating evidence suggests that psychological factors can have a negative effect on disease outcomes in dermatological conditions.^40^, ^41^, ^42^, ^43^ However, further prospective research into the predictive value of psychological factors for a broad range of skin conditions is needed to guide implementation of psychological markers in clinical practice. The SKINERGY study is designed to address this need by evaluating these factors and their potential clinical utility.

Several international initiatives are actively integrating multi‐omics strategies for deep phenotyping different patient cohorts (Table 1). The ImmUniverse consortium aims to improve the diagnosis, treatment and understanding of ulcerative colitis (UC) and AD by integrating patients' clinical, microbiome and transcriptomic data.^21^ In addition, BIOMAP evaluated genomics, transcriptomics, methylomics, proteomics and microbiomics to achieve a deep phenotyping of patients with AD and PSO.^22^ Furthermore, the HIPPOCRATES research programme focuses on identifying key molecular pathways in patients with psoriatic arthritis, using a comprehensive multi‐omics approach.^23^ A different consortium, that is, The Translational Dermatology Initiative, aims at a new disease classification of inflammatory skin diseases with repetitive multi‐omics analysis of tissue and integration of deep clinical phenotyping.^24^ All these initiatives have in common to create deep, high‐quality datasets optimized for machine learning. These projects are significantly improving our understanding of the application of multi‐omics techniques in deep phenotyping. However, the true synergy is anticipated to come from alignment of the cohorts for (i) external validation of different geographical spaces pan‐Europe and globally, (ii) larger sample sizes yielding increased statistical power, (iii) a broader population diversity leading to more robust findings and higher generalizability, (iv) cross‐comparison of different diseases and cohorts, (v) harmonization of protocols, designs and methodologies and ultimately establishing platforms and infrastructure to readily perform biomarker‐driven real‐world evidence studies in immuno‐dermatology.

**TABLE 1:** Overview of SKINERGY characteristics compared to other deep phenotyping consortia and projects.

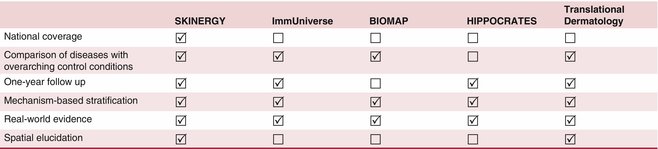	

**TABLE 2:** Overview of parameters as part of the multi‐omics assessments (various clinical assessments, imaging techniques, biophysical measurements, questionnaires and multi‐omics analyses) conducted at different timepoints (M0, M3, M6, M12) in patients with inflammatory skin diseases.

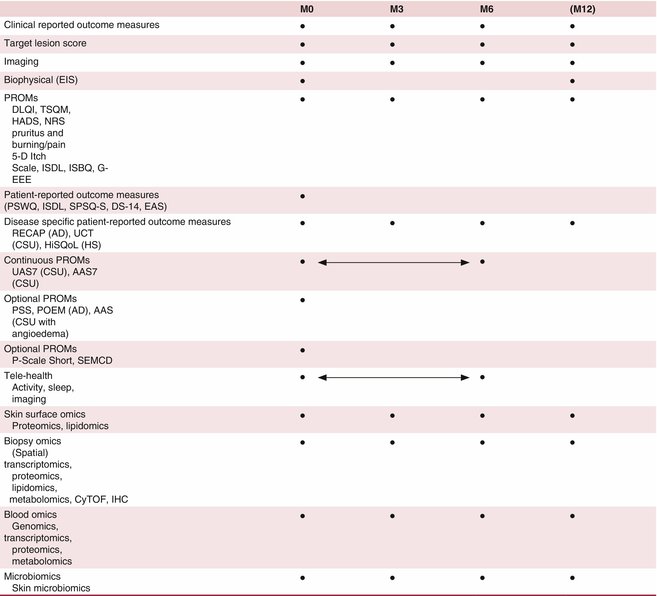	

*Note*: Data on continuous questionnaires and tele‐health monitoring are collected throughout the study period. Abbreviations: AAS7, Angioedema Activity Score over 7 days; AAS, Angioedema Activity Score; DLQI, Dermatology Life Quality Index; DS14, Type D Scale 14; EAS, Expectancy Avoidance Custom Scales; G‐EEE, Generic rating scale for previous treatment experiences, treatment expectations and treatment effects; HADS, Hospital Anxiety and Depression Scale; HiSQoL, Hidradenitis Suppurativa Quality of Life; ISBQ, Internalized Skin Bias Questionnaire; ISDL, Impact of Skin Disease on Daily Life; NRS, Numeric Rating Scale; POEM, Patient‐Oriented Eczema Measure; P‐Scale Short, Participation Scale Short version; PSS, Perceived Stress Scale; PSWQ, Penn State Worry Questionnaire; RECAP, Recap of Atopic Eczema; SEMCD, Self‐Efficacy to Manage Chronic Disease; SPSQ‐S, Sensory Processing Sensitivity Questionnaire Short version; TSQM, Treatment Satisfaction Questionnaire for Medication; UAS7, Urticaria Activity Score; UCT, Urticaria Control Test.

The SKINERGY study offers a unique and comprehensive research framework that sets it apart from other initiatives. With nationwide coverage across the Netherlands in all University Medical Centers, SKINERGY ensures broad and representative patient inclusion. A key strength is its cross‐disease comparative design, enabling the parallel study of multiple inflammatory skin diseases alongside overarching control groups to identify both shared and disease‐specific mechanisms. The one‐year longitudinal follow‐up design of the study allows for repetitive, detailed assessment of disease trajectories and treatment responses over time. Furthermore, SKINERGY applies mechanism‐based stratification, supporting a move towards biologically informed patient subgroups rather than relying solely on clinical phenotypes. By integrating real‐world observations from routine clinical care and incorporating spatially resolved analyses of skin tissue, frequent assessments at home, SKINERGY aims to deliver both high‐resolution and real‐life insights into disease pathogenesis. An additional strength is the incorporation of an external control cohort, predominantly malignant but also encompassing overlapping inflammation‐driven biology, which broadens the study's capacity for cross‐disease comparisons and improves the interpretability of immune pathway analyses.

A potential limitation is that small intergroup differences may limit the detection of statistically significant effects, although the cohort was designed to ensure a sufficient number of non‐responders for biomarker qualification. An additional limitation of this study is that participants are permitted to use topical medications for their skin condition, along with other concomitant treatments, which could influence the results. As the study design is non‐randomized, the bias of the treatment guidelines will influence the data, which is a clear limitation. However, despite these limitations, the choice of a real‐world study design is supported by its relevance and applicability to routine clinical practice. In addition, the synergy to concurrent ongoing consortia in other geographic regions is of pivotal importance for robustness, external validity and statistical power. ^21^, ^23^, ^24^, ^43^

In conclusion, the SKINERGY study aims to generate high‐quality, multi‐omics data on changes across various biomarkers. The biomarkers identified in this study have the potential to enable personalized treatment strategies in clinical practice and deepen the understanding of disease pathology in patients with AD, PSO, HS, CSU, CLE and MF. These advancements have the potential to significantly improve therapeutic efficacy and the quality of life for individuals with immune‐mediated inflammatory skin diseases and MF. Ultimately, we strive to overcome the ‘one‐size‐fits‐all’ approach and shift towards 'the right treatment, for the right patient, at the right time’.

## AUTHOR CONTRIBUTIONS

**N.G. Koster:** writing—original draft preparation; **J.M.P.A. van den Reek, E.M.G.J. de Jong, S. van Beugen, A.I.M. van Laarhoven, I. Haeck, M. de Bruin‐Weller, M.L. Schuttelaar, B. Horvath, A. Gostyński, A. Knulst, M.H. Vermeer, L. Nguyen, L. Gerbens, P. Spuls, M.A. Middelkamp‐Hup, V. Exadaktylos, T. Niemeyer‐van der Kolk, A.R. Schrader, A.M. Sluijmers, J. Versteeg, B.W.M. Arents, I. van Ee, J.L.A.G. van der Zon, M. de Leeuw, T. de Leeuw, P. van den Broek, C. Berkhof, D. Vellinga, S. Weidinger, S. Dubrak, Y. Li, M. van Steensel, M. Marchetti‐Deschmann, M. Maurer †, H. van der Zee, J. Damman, D.J. Hijnen, F. Wijk, M.M.B. Seyger, H. Röckmann, D.M.W. Balak and M.B.A. van Doorn:** conceptualization and methodology; **R. Rissmann:** conceptualization, methodology and supervision.

## FUNDING INFORMATION

This project is funded by the Dutch Research Council (NWO) under NWA‐ORC project NWA.1389.20.182 entitled Next Generation ImmunoDermatology.

## CONFLICT OF INTEREST STATEMENT

J.M.P.A. van den Reek has carried out clinical trials that were sponsored by Leo Pharma, Lilly, AbbVie, UCB and Janssen. She has received payment or honoraria for lectures, presentations, speakers bureaus, manuscript writing or educational events from Lilly, Leo Pharma, Zoetis, AbbVie, Janssen and Almirall. She has received support for attending meetings and/or travel from Janssen. She has participated on a Data Safety Monitoring Board or Advisory board for Bristol Myers Squibb and Leo Pharma. She has held a leadership or fiduciary role in the International Psoriasis Council and Psoriasis patiënten Nederland. E.M.G.J. de Jong received research grants for the department of dermatology of the Radboud University Medical Center Nijmegen, the Netherlands from AbbVie, Almirall, BMS, Janssen Pharmaceutica, Leo Pharma, Lilly, Novartis and UCB, ZonMw, KCE, NWO, the National Psoriasis Foundation USA, RUMC/VGZ and RUMC/SMK. She has acted as a consultant and/or paid speaker for and/or participated in research sponsored by companies that manufacture drugs used for the treatment of psoriasis or atopic dermatitis, including AbbVie, Amgen, Almirall, Boehringer‐Ingelheim, Bristol‐Myers Squibb, Celgene, Galapagos, Janssen Pharmaceutica, Leo Pharma, Lilly, Novartis, Sanofi and UCB. All funding is not personal but goes to the Institution Radboud University Medical Center Nijmegen, the Netherlands. L.A.A. Gerbens is one of the main investigators of the TREatment of ATopic eczema, the Netherlands/Belgium registry (TREAT NL/BE), which receives departmental independent research grants from pharmaceutical industries since December 2019. She is a member of the TREAT Registry Taskforce Executive Committee, which receives research grants from pharmaceutical industries. Additionally, she is the project lead of the governmental funded UPDATE trial. P. Spuls is chief investigator (CI) of the systemic and phototherapy atopic eczema registry (TREAT NL/BE) for adults and children and receives departmental independent research grants for TREAT NL/BE registry from Pharma since December 2019. She is member of the TREAT Registry Taskforce (TrT) Executive Committee. The TrT receive research grants for specific projects to combine data of different national registries. She is involved in performing clinical trials with many pharmaceutical industries that manufacture drugs used for the treatment of for example, psoriasis and atopic dermatitis, for which financial compensation is paid to the department/hospital. She was involved in the development of one of the HOME core outcome instruments (Recap of atopic eczema (RECAP)), and in the development of the Outcome Measures for VAscular Malformations (OVAMA) questionnaire for vascular malformations. Additionally, the is project lead of the governmental funded UPDATE trial to investigate NB‐UVB in atopic dermatitis. She has recently done a consultancy for Takeda (2025). I. van Ee is a volunteer patient representative for Psoriasis Patiënten Nederland and serves as an IFPA Ambassador. In this capacity, they bring the patient perspective to various projects focused on life with psoriasis and the care for people living with psoriasis or asked as a speaker. Some of these projects receive funding from pharmaceutical companies, including AbbVie, Johnson, Novartis, BMS, Almirall, UCB, Boehringer Ingelheim and LEO Pharma. All funding is provided to the respective organizations (Psoriasis Patiënten Nederland and IFPA), and not received personally and are unrelated to the present manuscript. J.L.A.G. van der Zon is a volunteer patiënt representative for Psoriasispatienten Nederland. In this capacity, he brings the patient perspective to various projects focused on life with psoriasis and psoriatic arthritis and the care for people living with psoriasis or is asked as a speaker. Some of these projects receive funding from pharmaceutical companies, including Johnson & Johnson and BMS. All funding is provided to the respective organizations (Psoriasispatienten Nederland and not received personally and are unrelated to the present manuscript). S. Weidinger received grants or contracts from Pfizer and Sanofi, of which payments were made to the institution. He received consulting feed from AbbVie, Almirall, Astria, Boehringer, Galderma, GSK, Leo Pharma, Pfizer, Regeneron Pharmaceuticals and Sanofi. He received payment or honoraria for lectures, presentations, speakers bureaus, manuscript writing or educational events from AbbVie, Almirall, Leo Pharma, Pfizer, Regeneron Pharmaceuticals and Sanofi. He received support for attending meetings and/or travel from AbbVie, Almirall, Leo Pharma, Pfizer and Sanofi. M. Maurer has received grants from Alexion, Allakos, Almirall, Amgen, AstraZeneca, Celldex, Clinuvel, Escient, Evommune, GSK, Incyte, Jasper, Kyowa Kirin, Leo Pharma, Lilly, Mitsubishi Tanabe Pharma, Moxie, Noucor, Novartis, Sanofi/Regeneron, Santa Ana Bio, Septerna, Servier, Teva, Third HarmonicBio, ValenzaBio and Yuhan Corporation. He has received fees for speaking or serving as an advisor from Allakos, Alvotech, Amgen, Aquestive, argenX, AstraZeneca, Celldex, Celltrion, Escient, Evommune, Excellergy, GSK, Incyte, Jasper, Kashiv, Lilly, Menarini, Mitsubishi Tanabe Pharma, Moxie, Noucor, Novartis, Orion Biotechnology, Resonance Medicine, Sanofi/Regeneron, Santa Ana Bio, Septerna, Servier, Teva, Third HarmonicBio, ValenzaBio, Vitalli Bio, Yuhan Corporation and Zurabio. H. van der Zee has received a grant or contract from UCB. Additionally, he has received consulting fees from UCB and Novartis. D.J. Hijnen received grants or contracts from clinical trials with AbbVie, Sanofi, Galderma and Almirall. There was registry support from Pfizer and Lilly. All funding is not personal but goes to the independent research fund of the department of dermatology of Radboudumc Nijmegen, the Netherlands. He received payment or honoraria for lectures, presentations, speakers bureaus, manuscript writing or educational events, of which all funding went to charity. F. van Wijk received grants or contracts from Leo Pharma, Sanofi, Almirall, Galapagos, Bristol‐Myers Squibb and AbbVie. She reeived consulting fees from Calypso Biotech. All payments were made to institution, and all grants are unrelated to the present manuscript. M.M.B. Seyger has received consulting fees from AbbVie, Bristol‐Myers Squibb, Eli Lilly, Janssen, Novartis and UCB. She has participated on a Data Safety Monitoring Board or Advisory Board for AbbVie, Bristol‐Myers Squibb and Janssen. All funding is not personal but goes to the independent research fund of the department of dermatology of Radboudumc Nijmegen, the Netherlands. H. Röckmann has received research funding from Novartis to support this work. She received institutional sponsoring for research, consultancy or speakers fee outside the submitted work from Pharming and Novartis Pharma, Sanofi, Third Harmonic Bio, AbbVie and Leo Pharma. D.M.W. Balak has participated in an advisory board of AbbVie. M.B.A. van Doorn is or recently was a speaker and/or advisor for and/or has received research funding from Novartis, AbbVie, Almirall, Pfizer, LEO Pharma, Sanofi, Lilly, Janssen, UCB, BMS, GSK, Maruho, Celgene, Third HarmonicBio and Escient. All other authors state no Conflict of Interest.

## TRIAL REGISTRIES

Clinicaltrial.gov NCT07021495; ABR NL88073.058.24.

## ETHICAL APPROVAL

Reviewed and approved by Medical Ethics Committee Leiden The Hague Delft, number P24.100.

## ETHICS STATEMENT

Patients provide written informed consent for the publication of their case details prior to participation. No patients have been enrolled at the time of manuscript submission.

## Supporting information


Data S1.

## Data Availability

The data that support the findings of this study are available from the corresponding author upon reasonable request.
