# Supplementary material for: Multi‐omics profiling of chronic immune‐mediated skin diseases: SKINERGY protocol and strategic evaluation
Source: J Eur Acad Dermatol Venereol. 2026 Feb 6;40(9):1490–501. doi: 10.1111/jdv.70311 (PMC13505733; doi:10.1111/jdv.70311)
Supplement: Supplementary file 1 [file JDV-40-1490-s001.docx]

Supplemental information pertaining to:

Multi-omics profiling of chronic, immune-mediated inflammatory skin diseases: Study protocol for the nationwide, prospective biomarker study SKINERGY in the Netherlands

N.G. Koster, et al.

**Protocol main body**

Inclusion criteria

All patients must meet all of the following inclusion criteria for study entry:

1. Able to understand and provide a written informed consent prior to any study procedures
2. Male or non-pregnant female, ≥18 years of age
3. Patient is willing to refrain from extensively washing (including bathing, swimming) the target lesional skin 12 hours before every study visit day.
4. Patient is willing and able to comply with the study protocol
5. Female participants are willing to not get pregnant between M0 until M12, from study entry to the last study visit
6. The patient is willing to start the prescribed treatment.

*Disease-specific inclusion criteria*

For patients with AD:

7. Diagnosis and history of chronic, moderate-to-severe AD (by the Eichenfield revised criteria of Hanifin and Rajka for at least 3 years before baseline visit.
8. Documented recent history (last 6 months) of eligibility for (local or systemic) treatment with immunosuppressants, biologics or JAK-inhibitors.
9. When applicable, documented recent history (last 6 months) of inadequate response to treatment with topical therapy, immunosuppressants, biologics or JAK-inhibitors.
10. Current treatment can include moisturizers, topical treatment and/or systemic treatments with preferable wash-out (see exclusion criterion #9). On-study treatment is at physician and patient discretion but must include eligibility to starting new systemic treatment.
11. EASI≥7 (moderate-to-severe disease)
12. At least one suitable target lesion at the discretion of the investigator
13. Intention to start treatment with cyclosporine A, dupilumab, tralokinumab, lebrikizumab or a JAK1-inhibitor (abrocitinib or upadacitinib)

For patients with CLE:

Participants must have a diagnosis of CLE, including SCLE, CDLE or LET that fulfil the following:

7. Confirmed CLE diagnosis by clinicopathological correlation.
8. An overall CLE Disease Area and Severity Index Activity (CLASI-A) Score ≥3 without counting any diffuse alopecia or oral ulcers.
9. Intention to start treatment with TCS, hydroxychloroquine or methotrexate (combination or mono-treatment).

For patients with CSU:

7. Diagnosis of CSU (moderate to severe according to international guidelines (Zuberbier et al, 2022)) for ≥3 months and symptomatic disease despite treatment with second generation H1 antihistamines (up to fourfold the approved dose).
8. Patients currently on an antihistamine (up to fourfold the approved dose) must be on a stable dose for at least 2 weeks prior to day 1 and must maintain the same stable dose throughout the treatment period.
9. Intention to start (add-on to antihistamine) treatment of omalizumab, cyclosporine A or BTK inhibitor*. (*when approved and reimbursed in NL)

For patients with HS:

7. Patient with a history of signs and symptoms consistent with moderate-to-severe HS, based on IHS4 score (Zouboulis et al., 2017), for at least 1 year prior to baseline
8. Current treatment can include topical treatment. On-study treatment is at physician and patient discretion but must include eligibility to starting systemic treatment
9. Intention to start treatment with anti-TNF or anti-IL17 (secukinumab, bimekizumab*) *when approved and reimbursed in NL.

For patients with MF:

7. A confirmed diagnosis of CTCL MF type and stage classification via histology or clinicopathological correlation
8. For the stage IA-IIA CTCL patients: at least one patch and/or one plaque lesion is present
9. Intention to start treatment with topical chlormethine, topical corticosteroids or phototherapy (PUVA / UV-B).

For patients with PSO:

7. Diagnosed with chronic plaque psoriasis at least 6 months prior to study participation
8. PASI≥5 with at least one suitable target lesion at the discretion of the investigator
9. Current treatment can include moisturizers, topical treatment and/or systemic treatments with preferable wash-out. On-study treatment is at physician and patient discretion but must include eligibility to starting new systemic treatment
10. Intention to start treatment with biologics: anti-TNF, anti-IL23, anti-IL17 or anti-TYK2

For healthy volunteers:

1. Signed informed consent before any study-mandated procedure.
2. Male or non-pregnant female volunteers, $\geq$ 18 years of age
3. Subject is in stable good health as per judgement of the investigator based upon the results of medical history and assessments performed at baseline.
4. No clinically significant skin disease as judged by the investigator.
5. No history of hypertrophic scarring or keloid.
6. Subject is willing to refrain from extensively washing (including bathing, swimming) the skin 12 hours before every study visit.
7. Subject is willing and able to wash out and withhold any topical treatment (prescription and over-the-counter products) in the investigational area for 2 weeks prior to Day 1.
8. Subject is willing to refrain from application of any topical product (e.g. ointments, cream, or washing lotions) on the skin 24 hours prior to every study visit day.
9. Subject is willing and able to wash out any antibiotic therapy for 14 days prior to Day 1.
10. Subject is willing and able to comply with the study protocol.
11. Female participants are willing to not get pregnant from study entry to the last study visit

Exclusion criteria

Eligible patients must not meet the following exclusion criteria:

1. Have any other relevant skin infection/disease in the treatment area other than the investigated skin disease.
2. Subjects who have received treatment with any non-marketed drug substance (that is, an agent which has not yet been made available for clinical use following registration) within 4 weeks prior to the baseline visit.
3. Any other condition, disease, or known factor that could interfere with the study conduct or the study objectives as per judgement of the investigator.
4. Having received treatments for the investigated skin disease within the following intervals prior to the start of the study is not a strict exclusion criterion since this is a real-world study. However, preferred intervals for washout are as follows:
   a. 1 week for topical treatment, e.g. corticosteroids, retinoids, vitamin D analogs, calcineurin inhibitors
   b. 4 weeks for phototherapy, e.g. UVB, PUVA, PDT
   c. 4 weeks for non-biologic systemic treatment, e.g. retinoids, methotrexate, cyclosporine, JAK inhibitors
   d. 8 weeks for radiotherapy or surgery in the treatment area
   e. 8 weeks for biologics
   f. 3 months for any systemic chemotherapeutical treatment

*Disease specific exclusion criteria*

For patients with CLE:

5. Diagnosed with SLE

For patients with CSU:

5. Treatment with omalizumab within 8 weeks prior to Day 1
6. Urticarial or angioedema symptoms such as urticarial vasculitis, erythema multiforme, cutaneous mastocytosis (urticaria pigmentosa) and hereditary, acquired angioedema or drug-induced (e.g., due to C1 esterase inhibitor deficiency, ACE-inhibitor induced).

For patients with MF:

5. Ongoing uncontrolled active skin infection, other than secondary impetiginized CTCL lesions as judged by the investigator

For patients with PSO:

5. Having primarily erythrodermic, pustular or guttate psoriasis;
6. Having drug-induced psoriasis;

For healthy volunteers:

1. History of immunological abnormality (e.g. immune suppression, severe allergy, or anaphylaxis) that may interfere with study objectives as per judgement of the investigator.
2. History or symptoms of any uncontrolled, significant disease including (but not limited to), a neurological, psychiatric, endocrine, cardiovascular, respiratory, gastrointestinal, hepatic, or renal disorder that may interfere with the study objectives as per judgement of the investigator.
3. The use of systemic antibiotic therapy for >2 months in the past 12 months.
4. The use of any immunosuppressive or immunomodulatory therapy within the past 30 days prior to Day 1.
5. Loss or donation of blood over 500mL within three months prior to baseline. Participation in an investigational drug study within 3 months prior to baseline visit or more than 4 times a year.
6. History of alcohol consumption exceeding 5 standard drinks per day on average within 3 months prior to baseline. Alcohol consumption will be prohibited for at least 24 hours preceding each study visit.
7. Positive urine test for drugs or history of abuse at baseline.
8. Exposure to high doses of UV radiation is not permitted within 3 weeks of the first study visit until the end of the study
9. Extreme physical activities are not permitted within 48 hours before each study visit
10. Any other condition, disease, or known factor that could interfere with the study conduct or the study objectives as per judgement of the investigator.

Objectives

The protocol states the following primary objectives:

- To identify discriminative biomarkers for predictivity of treatment (non)response
- To evaluate disease-related biomarkers compared to healthy volunteers
- To evaluate (multi-omics) biomarkers for disease stratification and (targeted) treatment (non)response in daily practice of patients with AD, CLE, CSU, HS, MF and PSO

Secondary objectives include:

- To compare biomarkers between different patient cohorts
- To evaluate changes in biomarkers upon treatment over time

Exploratory objectives include:

- To identify biomarkers in skin tissue that correlate with disease progression or therapy response
- To evaluate and compare genome profiles of patients
- To include additional (imaging) biomarkers in comparison of profiles and over time

Response definitions

The response levels are defined per disease-specific criteria and are as follows: For AD, EASI-based response levels are classified as non-response (<25% improvement), some response (≥25%), good response (≥50%), very good response (≥75%), complete response (≥90%), and super response (100% at week 12). A clinically meaningful response is defined as achieving EASI-50, a ≥3-point reduction in peak pruritus NRS, or a ≥4-point improvement in DLQI at week 16. Additional criteria include a ≥8-point improvement in POEM, a ≥4-point improvement in DLQI, a ≥3-point reduction in itch NRS, and ≥70% treatment satisfaction on the TSQM. For PSO, similar PASI-based thresholds are applied. In HS, a good response is defined as a ≥55% reduction in IHS4 score. In MF, response categories range from complete response (100% clearance) to partial response (50–99% clearance), stable disease, progressive disease, and relapse, according to Olsen et al. (2011). For CSU, response is classified by UAS7: non-response (>16), partial response (7–15), good response (1–6), and complete response (0), with response speed further categorized as fast (4–6 weeks) or slow (12–16 weeks). In CLE, response is defined by CLASI-A improvement: non-response (<20%), some response (≥20%), good response (≥50%), very good response (≥75%), complete response (≥90%), and super response (100%).

**Strengthening the Reporting of Observational studies in Epidemiology (STROBE) checklist**

|  | Item No | Recommendation |
| --- | --- | --- |
| **Title and abstract** | 1 | (*a*) Indicate the study’s design with a commonly used term in the title or the abstract 🗹 |
|  |  | (*b*) Provide in the abstract an informative and balanced summary of what was done and what was found 🗹 |
| Introduction | | |
| Background/rationale | 2 | Explain the scientific background and rationale for the investigation being reported 🗹 |
| Objectives | 3 | State specific objectives, including any prespecified hypotheses 🗹 |
| Methods | | |
| Study design | 4 | Present key elements of study design early in the paper 🗹 |
| Setting | 5 | Describe the setting, locations, and relevant dates, including periods of recruitment, exposure, follow-up, and data collection 🗹 |
| Participants | 6 | (*a*) Give the eligibility criteria, and the sources and methods of selection of participants. Describe methods of follow-up 🗹 |
|  |  | (*b*) For matched studies, give matching criteria and number of exposed and unexposed N/A |
| Variables | 7 | Clearly define all outcomes, exposures, predictors, potential confounders, and effect modifiers. Give diagnostic criteria, if applicable 🗹 |
| Data sources/ measurement | 8* | For each variable of interest, give sources of data and details of methods of assessment (measurement). Describe comparability of assessment methods if there is more than one group 🗹 |
| Bias | 9 | Describe any efforts to address potential sources of bias 🗹 |
| Study size | 10 | Explain how the study size was arrived at 🗹 |
| Quantitative variables | 11 | Explain how quantitative variables were handled in the analyses. If applicable, describe which groupings were chosen and why 🗹 |
| Statistical methods | 12 | (*a*) Describe all statistical methods, including those used to control for confounding 🗹 |
|  |  | (*b*) Describe any methods used to examine subgroups and interactions 🗹 |
|  |  | (*c*) Explain how missing data were addressed ⮽ |
|  |  | (*d*) If applicable, explain how loss to follow-up was addressed ⮽ |
|  |  | (*e*) Describe any sensitivity analyses ⮽ |
| Results | | |
| Participants | 13* | (a) Report numbers of individuals at each stage of study—eg numbers potentially eligible, examined for eligibility, confirmed eligible, included in the study, completing follow-up, and analysed N/A |
|  |  | (b) Give reasons for non-participation at each stage N/A |
|  |  | (c) Consider use of a flow diagram N/A |
| Descriptive data | 14* | (a) Give characteristics of study participants (eg demographic, clinical, social) and information on exposures and potential confounders N/A |
|  |  | (b) Indicate number of participants with missing data for each variable of interest N/A |
|  |  | (c) Summarise follow-up time (eg, average and total amount) N/A |
| Outcome data | 15* | Report numbers of outcome events or summary measures over time N/A |
| Main results | 16 | (*a*) Give unadjusted estimates and, if applicable, confounder-adjusted estimates and their precision (eg, 95% confidence interval). Make clear which confounders were adjusted for and why they were included N/A |
|  |  | (*b*) Report category boundaries when continuous variables were categorized N/A |
|  |  | (*c*) If relevant, consider translating estimates of relative risk into absolute risk for a meaningful time period N/A |
| Other analyses | 17 | Report other analyses done—eg analyses of subgroups and interactions, and sensitivity analyses N/A |
| Discussion | | |
| Key results | 18 | Summarise key results with reference to study objectives N/A |
| Limitations | 19 | Discuss limitations of the study, taking into account sources of potential bias or imprecision. Discuss both direction and magnitude of any potential bias 🗹 |
| Interpretation | 20 | Give a cautious overall interpretation of results considering objectives, limitations, multiplicity of analyses, results from similar studies, and other relevant evidence N/A |
| Generalisability | 21 | Discuss the generalisability (external validity) of the study results N/A |
| Other information | | |
| Funding | 22 | Give the source of funding and the role of the funders for the present study and, if applicable, for the original study on which the present article is based 🗹 |

**List of non-standard abbreviations**

AD	Atopic Dermatitis

anti-BTK	Anti-Bruton's Tyrosine Kinase

anti-IgE	Anti-Immunoglobulin E

anti-IL4/13	Anti-Interleukin 4/13

anti-IL17	Anti-Interleukin 17

anti-IL23	Anti-Interleukin 23

anti-JAK	Anti-Janus Kinase

anti-TNF	Anti-Tumor Necrosis Factor

anti-TYK2	Anti-Tyrosine Kinase 2

CHLORM	Chlormethine

CLE	Cutaneous Lupus Erythematosus

CsA	Cyclosporine A

CSU	Chronic Spontaneous Urticaria

CyTOF	Imaging Mass Cytometry

EDC	Electronic Data Capture

EpiTOF	Cytometry by Time-Of-Flight

ePRO	Patient Reported Outcome

HCQ	Hydroxychloroquine

HS	Hidradenitis Suppurativa

MF	Mycosis fungoides-type cutaneous T-cell lymphoma

MTX	Methotrexate

NGID	Next Generation ImmunoDermatology

PROMs	Patient-reported outcome measures

PSO	Psoriasis

PUVA/UV-B	Psoralen + Ultraviolet A / Ultraviolet B

TSC	Topical Corticosteroids

UC	Ulcerative Colitis

**Supplementary tables**

**Table S1. Overview of all Physician Reported Outcome Measures in the SKINERGY study**

| Physician Reported Outcome Measure | Abbreviation | Indication | Reference |
| --- | --- | --- | --- |
| Eczema Area and Severity Index | EASI | AD | (Hanifin et al., 2001) |
| Validated Investigator Global Assessment for Atopic Dermatitis | vIGA-AD | AD | (Simpson et al., 2020) |
| oSCORAD | - | AD | (Oranje et al., 2007) |
| Psoriasis Area and Severity Index | PASI | PSO | (Robinson et al., 2012) |
| Physician Global Assessment | PGA | PSO | (Robinson et al., 2012) |
| Body Surface Area | BSA | PSO | (Bozek and Reich, 2017) |
| PASI-High Discrimination | PASI-HD | PSO | (Papp et al., 2021) |
| International Hidradenitis Suppurativa Severity Score System | IHS-4 | HS | (Zouboulis et al., 2017) |
| Body Surface Area | BSA | MF | (Novelli et al., 2016) |
| Composite Assessment of Index Lesion Severity | CAILS | MF | (Olsen et al., 2011) |
| Modified Severity-Weighted Assessment Tool | mSWAT | MF | (Gu et al., 2022) |
| Cutaneous Lupus Activity Investigators Global Assessment | CLA-IGA | CLE | (Paolino et al., 2023) |
| Cutaneous Lupus Disease Area and Severity Index Area | CLASI-A | CLE | (Chakka et al., 2021) |

**Table S2. Overview of all PROMs in the SKINERGY study**

| PROM | Abbreviation | Reference |
| --- | --- | --- |
| Dermatology Life Quality Index | DLQI | (Finlay and Khan, 1994) |
| Treatment Satisfaction Questionnaire for Medication | TSQM | (Atkinson et al., 2004) |
| Hospital Anxiety and Depression Scale | HADS | (Spinhoven et al., 1997) |
| Numeric Rating Scale pruritus and pain/burning | NRS | (Yosipovitch et al., 2019) |
| 5-D itch scale | - | (Elman et al., 2010) |
| Impact of Chronic Skin Disease on Daily Life | ISDL | (Evers et al., 2008) |
| Sensory Processing Sensitivity | SPSQ-S | (De Gucht and Woestenburg, 2024) |
| Generic rating scale for previous treatment experiences, treatment expectations, and treatment effects | G-EEE | (Rief et al., 2021) |
| Custom skin condition- and itch-related Expectancies and Avoidance Scales | EAS | - |
| Penn State Worry Questionnaire | PSQW | (Meyer et al., 1990) |
| Internalized Stigma | ISBQ | (Butt et al., 2022) |
| Type D scale 14 personality | DS-14 | (Denollet, 2005) |
| Recap of atopic eczema (RECAP)* | RECAP | (Howells et al., 2020) |
| Hidradenitis Suppurativa Quality of Life* | HiSQOL | (Kirby et al., 2020; Thorlacius et al., 2019) |
| Urticaria Control Test* | UCT | (Ohanyan et al., 2017) |
| Urticaria Activity Score 7* | UAS7 | (Hawro et al., 2018) |
| User experience and subjective burden questionnaire** | - | - |
| Perceived Stress Scale** | PSS | (Cohen et al., 1983) |
| Social Participation Scale** | P-scale short | (Van Brakel et al., 2006) |
| Self-Efficacy for Managing Chronic Disease Scale** | SEMCD | (Ritter and Lorig, 2014) |
| Patient-Oriented Eczema Measure** | POEM | (Charman et al., 2004) |
| Angioedema Activity Score** | AAS | (Weller et al., 2013) |

* Disease-specific PROMs, to be completed if applicable
** Optional PROMs

**Table S3. Overview of multi-omics approach on skin surface, blood and biopsy (tissue) samples of lesional- and non-lesional skin of patients with inflammatory skin diseases**

|  | Skin surface (Lesional and non-lesional) | Blood | Biopsy (Lesional and non-lesional) |
| --- | --- | --- | --- |
| Genomics |  | • | Spatial |
| Transcriptomics |  | • | Spatial |
| Proteomics | • | • | Spatial |
| Lipidomics |  | • | Spatial |
| Metabolomics |  | • | Spatial |
| Imaging mass cytometry (CyTOF) |  |  | Spatial |
| Immunohistochemistry (IHC) |  |  | Spatial |
| Microbiomics | • |  |  |
